# Lower limb muscle strength, inter-limb asymmetry, and vertical jump performance in elite female football players: A cross-sectional study

**DOI:** 10.1371/journal.pone.0350952

**Published:** 2026-08-28

**Authors:** Danail Georgiev Ivanov, Petar Peev

**Affiliations:** National Sports Academy “Vassil Levski”, Sofia, Bulgaria; Portugal Football School, Portuguese Football Federation, PORTUGAL

## Abstract

Lower limb strength imbalance and inter-limb asymmetry are relevant neuromuscular screening markers in women’s football, yet data from national-champion squads in Eastern Europe remain limited. The aim of this study was to characterise lower limb isometric knee strength, hamstring-to-quadriceps (H:Q) ratios, and bilateral and unilateral squat jump (SJ) and countermovement jump (CMJ) performance, and to describe their distribution by playing position, in elite Bulgarian female football players. Nineteen players from FC NSA Sofia Women (Bulgarian Women’s Football Champions, 2024/25; age 20.9 ± 4.3 years; height 168.4 ± 4.7 cm; body mass 60.5 ± 8.0 kg; body mass index 21.3 ± 2.7 kg·m^−2^) were assessed on a single testing day using a handheld dynamometer (Kineo Intelligent Load) and an optical jump system (Optojump Next). Mean H:Q ratios were 0.58 ± 0.10 (left, 95% CI [0.52, 0.64]) and 0.55 ± 0.07 (right, 95% CI [0.51, 0.60]); the right-limb mean was statistically below the 0.60 reference threshold (t(13) = −2.40, p = 0.032, d = −0.64). Thirteen of fourteen assessed players (92.9%, 95% CI [68.5–98.7%]) presented at least one limb with an H:Q ratio below 0.60. Mean bilateral SJ and CMJ were both 29.2 cm, producing a near-zero elasticity index (0.99 ± 10.6%). Unilateral asymmetry exceeding 10% was observed in 52.9% of players for SJ (95% CI [31.0–73.8%]) and 35.3% for CMJ (95% CI [17.3–58.7%]). Positional profiles are reported descriptively only, given the small subgroup sizes. The findings support routine neuromuscular screening of lower limb strength balance and unilateral jump performance in elite women’s football, and indicate that conditioning programmes should address both hamstring and quadriceps capacity rather than hamstring strength in isolation.

## Introduction

Women’s football has expanded rapidly over the past two decades, with increased participation, professionalisation, and physical demands across domestic and international competitions [1]. This development has intensified interest in neuromuscular screening approaches that can support performance development and injury-prevention strategies in female players.

Anterior cruciate ligament (ACL) injuries remain among the most severe injuries in women’s football, frequently resulting in prolonged absence from play, reduced career longevity, and post-injury changes in physical and psychological capacity [2,3]. Contemporary epidemiological and prospective injury surveillance studies in elite female football continue to report ACL injury rates substantially higher than those of male counterparts in comparable competitive contexts, with multifactorial anatomical, hormonal, biomechanical, and neuromuscular determinants [4,5]. Although injury risk cannot be inferred directly from cross-sectional screening alone, lower limb strength balance and inter-limb asymmetry are commonly examined because they may identify modifiable neuromuscular characteristics relevant to individualised training prescription and return-to-play decision-making [6,7].

The hamstring-to-quadriceps (H:Q) strength ratio is frequently used to describe the balance between knee flexor and extensor strength. Lower H:Q ratios may reflect relative quadriceps dominance and reduced hamstring capacity to resist anterior tibial translation and to support knee joint control during sprinting, landing, deceleration, and change-of-direction actions [8,9]. In applied strength and conditioning settings, an H:Q ratio of approximately 0.60 is often used as a reference threshold, although the interpretation depends on testing mode, joint angle, contraction type, and the specific population assessed [6].

Inter-limb asymmetry is another relevant screening construct. Asymmetry can be quantified during strength tests or unilateral jump tasks, and values greater than 10% are often considered practically meaningful in applied contexts [8,9]. Squat jump (SJ) and countermovement jump (CMJ) tests provide feasible measures of lower limb explosive capacity and stretch-shortening cycle utilisation, with relevance for sprinting, acceleration, braking, and aerial duels in football [10,11].

Physical profiles in football are also influenced by playing position. Forwards and wingers may rely heavily on acceleration and explosive actions, midfielders may accumulate high volumes of repeated locomotor actions, and defenders frequently perform deceleration, tackling, and aerial duels [12,13]. However, position-specific neuromuscular screening data in elite women’s football, particularly in Eastern European national-champion squads, remain limited.

Therefore, the aims of this study were to: (1) characterise isometric knee extensor and flexor strength and H:Q ratios; (2) quantify bilateral and unilateral SJ and CMJ performance; (3) determine the prevalence of clinically meaningful inter-limb asymmetry; and (4) describe positional differences in these parameters in elite female football players from the Bulgarian national champions. Based on previous reports describing high prevalence of suboptimal H:Q ratios and meaningful unilateral asymmetry in female team-sport athletes, we hypothesised that (i) a substantial proportion of players would present H:Q ratios below the 0.60 reference threshold on at least one limb, and (ii) the prevalence of unilateral SJ or CMJ asymmetry exceeding 10% would be clinically relevant within this cohort. No directional hypothesis was specified for positional comparisons because subgroup sizes were anticipated to be small and these comparisons were planned as descriptive only.

## Materials and methods

### Ethics statement

The study protocol was reviewed and approved by the Scientific Ethics Committee of the National Sports Academy “Vassil Levski”, Sofia, Bulgaria (approval No. EC-NSA-2026-004; date: 24 April 2026). The study was conducted in accordance with the ethical principles of the Declaration of Helsinki. All participants were informed about the aims, procedures, potential risks, and voluntary nature of the study before data collection. Written informed consent was obtained from all participants before testing. For participants under 18 years of age, written informed consent was obtained from a parent or legal guardian, together with participant assent.

### Study design and participants

This was a cross-sectional observational study based on a whole-squad convenience sample. All available members of FC NSA Sofia Women, the Bulgarian Women’s Football Champions for the 2024/25 season, were invited to participate; the sample size (n = 19) therefore reflects the total roster of the championship squad at the time of testing rather than an a priori power calculation. No member of the squad declined participation. This sampling approach was selected because the study aimed to provide a descriptive neuromuscular profile of the national-champion squad as a whole, and because an a priori power calculation was not feasible given the absence of normative reference values for elite female football players in this national context. Participant recruitment and all data collection were conducted on a single testing day, on 27 April 2026. The cohort characteristics were: age 20.9 ± 4.3 years (range 16–33 years), height 168.4 ± 4.7 cm (range 160–181 cm), body mass 60.5 ± 8.0 kg (range 50.0–75.0 kg), and body mass index (BMI) 21.3 ± 2.7 kg·m^−2^ (range 17.7–26.2 kg·m^−2^). Players were distributed across the following positional groups: goalkeeper (n = 1), fullback (n = 5), central defender (n = 2), midfielder (n = 5), winger (n = 3), and forward (n = 3). All participants were free of injury at the time of testing and had completed at least six months of structured club training before assessment. A clear age/body-mass transposition identified for one participant during data screening was treated as missing and excluded from the descriptive statistics for those two variables; her remaining data were retained. To control for known sources of acute variability in neuromuscular performance, participants were instructed to (i) maintain their habitual sleep schedule and to report at least seven hours of sleep on the night preceding the test; (ii) consume a standard pre-training meal at least two hours prior to testing and to avoid unfamiliar foods; (iii) abstain from caffeine, energy drinks, and other stimulants for the 24 hours preceding the test; and (iv) attend the testing session at the same time of day. Compliance with these instructions was confirmed verbally upon arrival.

### Anthropometric measurements

Body height was measured to the nearest 0.1 cm using a calibrated stadiometer. Body mass was recorded to the nearest 0.1 kg using a certified digital scale. Measurements were obtained under standardised morning conditions.

### Isometric muscle strength assessment

Maximal isometric knee extension and flexion forces were assessed bilaterally using a Kineo Intelligent Load dynamometer (Globus, Codognè, Italy). Participants were positioned with 90° hip flexion. Knee angle was set at 90° for flexion and 60° for extension assessment, consistent with standardised dynamometry protocols [10]. Three maximal voluntary isometric contractions were performed per limb and movement direction, with 30 s rest between trials. The highest valid force value was retained for analysis. The H:Q ratio was calculated independently for each limb as maximal knee flexion force divided by maximal knee extension force. Absolute strength asymmetry was calculated using the standard inter-limb percentage difference formula as |right - left| / max(right, left) × 100, in accordance with the recommended approach for inter-limb asymmetry in strength and conditioning practice [11]. The prevalence of strength asymmetry was evaluated using a >10% threshold.

### Jump performance assessment

Explosive lower limb performance was assessed using bilateral and unilateral SJ and CMJ protocols with the Optojump Next system (Microgate, Bolzano, Italy), a validated optical system for estimating vertical jump height from flight time [14]. Before testing, participants completed a 10 min standardised warm-up comprising light jogging, dynamic mobility exercises, and three submaximal practice jumps. For the SJ, participants descended to approximately 90° knee flexion, held the position for 2 s, and performed a maximal vertical jump without countermovement. For the CMJ, a self-selected countermovement depth was permitted. Unilateral trials were performed on each leg in a counterbalanced order. Three valid trials were recorded per condition, and the highest jump height was retained.

The elasticity index (EI) was calculated as [(CMJ - SJ) / SJ] × 100, in accordance with the classical Bosco protocol [12,13]. Directional unilateral jump asymmetry was calculated using the same standardised formula applied to strength data: [(right - left) / max(right, left)] × 100. Positive values indicate right-limb advantage and negative values indicate left-limb advantage. The prevalence of meaningful asymmetry was calculated from the absolute value of directional asymmetry using a >10% threshold, consistent with applied recommendations [11].

### Statistical analysis

Descriptive statistics are reported as mean ± standard deviation (SD), with minimum, maximum, coefficient of variation (CV%), and where appropriate 95% confidence intervals (CI) for means and proportions. Normality was assessed using the Shapiro-Wilk test, which is appropriate for sample sizes below 30. The prevalence of players exceeding clinical thresholds (H:Q < 0.60; absolute asymmetry > 10%) is expressed as absolute and relative frequencies with 95% Wilson score confidence intervals. Inter-limb differences for strength (knee extension, knee flexion, H:Q ratio) and unilateral jump performance (SJ, CMJ) were examined with paired-samples t-tests when the within-pair differences were approximately normally distributed, or Wilcoxon signed-rank tests otherwise. Effect sizes for paired comparisons were reported as Cohen’s dz, interpreted as small (≥0.20), moderate (≥0.50), or large (≥0.80). One-sample t-tests compared mean H:Q ratios against the 0.60 reference threshold, with Cohen’s d reported as the effect size. Positional-level data are reported descriptively only, without inferential between-group comparisons, because subgroup sizes (n = 1–5) were insufficient to support statistical between-group testing. Statistical significance was set at α = 0.05 (two-sided). All analyses were performed using IBM SPSS Statistics, version 26.0 (IBM Corp., Armonk, NY, USA).

## Results

### Anthropometric characteristics

Anthropometric and demographic characteristics are summarised in Table 1. The cohort showed relative homogeneity in stature (CV = 2.8%) and moderate variability in body mass (CV = 13.2%).

**Table 1:** Anthropometric and demographic characteristics of FC NSA Sofia Women (n = 19).

Variable	n	Mean ± SD	Min	Max	CV%	SW p-value
Age (years)	19	20.9 ± 4.3	16	33	20.4	>0.05
Height (cm)	19	168.4 ± 4.7	160	181	2.8	>0.05
Body mass (kg)	19	60.5 ± 8.0	50.0	75.0	13.2	>0.05

SD: standard deviation; CV%: coefficient of variation; SW: Shapiro-Wilk.

### Isometric knee strength and H:Q ratio

Isometric strength data for the 14 players with complete measurements are presented in Table 2. Mean knee extension strength was 37.2 ± 4.3 kg for the left limb and 39.1 ± 4.2 kg for the right limb. Mean H:Q ratios were 0.58 ± 0.10 and 0.55 ± 0.07 for the left and right limbs, respectively. Thirteen of 14 assessed players (92.9%) presented an H:Q ratio below 0.60 on at least one limb, and 75.0% of all individual limb observations fell below this threshold. Three players (21.4%) demonstrated inter-limb extension strength asymmetry exceeding 10%, with one player reaching 22.0%. Non-normal distributions were identified for left knee flexion (Shapiro-Wilk p = 0.018) and the right H:Q ratio (p = 0.008).

**Table 2:** Isometric lower limb muscle strength and H:Q ratio (n = 14).

Variable	Mean ± SD	Min	Max	CV%	SW p-value
Knee extension, left (kg)	37.2 ± 4.3	28.7	42.9	11.6	0.284
Knee extension, right (kg)	39.1 ± 4.2	30.7	45.1	10.6	0.437
Extension ASI (%)	5.2 ± 6.0	0.7	22.0	–	–
Knee flexion, left (kg)	21.7 ± 5.2	14.1	32.0	23.9	0.018*
Knee flexion, right (kg)	21.6 ± 3.4	16.7	27.0	15.8	0.771
Flexion ASI (%)	8.5 ± 6.5	0.5	22.2	–	–
H:Q ratio, left	0.58 ± 0.10	0.42	0.82	17.5	0.284
H:Q ratio, right	0.55 ± 0.07	0.41	0.67	12.9	0.008*

SD: standard deviation; CV%: coefficient of variation; ASI: asymmetry index; H:Q: hamstring-to-quadriceps ratio; SW: Shapiro-Wilk; *p < 0.05 indicates non-normal distribution.

### Jump performance

Bilateral and unilateral jump performance data are presented in Table 3. Mean bilateral SJ and CMJ were nearly equivalent (29.2 ± 4.0 cm and 29.2 ± 3.7 cm), yielding a near-zero mean elasticity index (0.99 ± 10.6%). Right limb jump height was marginally higher than left limb jump height for both SJ (15.1 ± 1.7 vs. 14.2 ± 1.7 cm) and CMJ (15.2 ± 1.8 vs. 14.4 ± 2.2 cm). Mean directional unilateral asymmetry was 5.9 ± 9.9% for SJ and 5.2 ± 11.4% for CMJ. Nine players (52.9%) exceeded the 10% asymmetry threshold for unilateral SJ, and six players (35.3%) exceeded the threshold for unilateral CMJ.

**Table 3:** Bilateral and unilateral squat jump (SJ) and countermovement jump (CMJ) performance (n = 17).

Variable	Mean ± SD	Min	Max	CV%	SW p-value
SJ bilateral (cm)	29.2 ± 4.0	21.8	35.3	13.8	0.764
CMJ bilateral (cm)	29.2 ± 3.7	23.6	36.8	12.5	0.733
Elasticity index (%)	0.99 ± 10.6	−21.1	19.1	–	0.936
SJ right (cm)	15.1 ± 1.7	12.3	18.5	11.4	0.497
SJ left (cm)	14.2 ± 1.7	10.2	17.7	11.9	0.551
SJ ASI (%)	5.9 ± 9.9	−10.4	23.3	–	0.783
CMJ right (cm)	15.2 ± 1.8	12.3	19.1	11.7	0.606
CMJ left (cm)	14.4 ± 2.2	8.7	18.6	15.4	0.434
CMJ ASI (%)	5.2 ± 11.4	−8.5	36.0	–	0.067

SD: standard deviation; CV%: coefficient of variation; ASI: asymmetry index. Positive ASI values indicate right-limb advantage; negative values indicate left-limb advantage. SW: Shapiro-Wilk.

### Positional comparison

**Fig 1:**
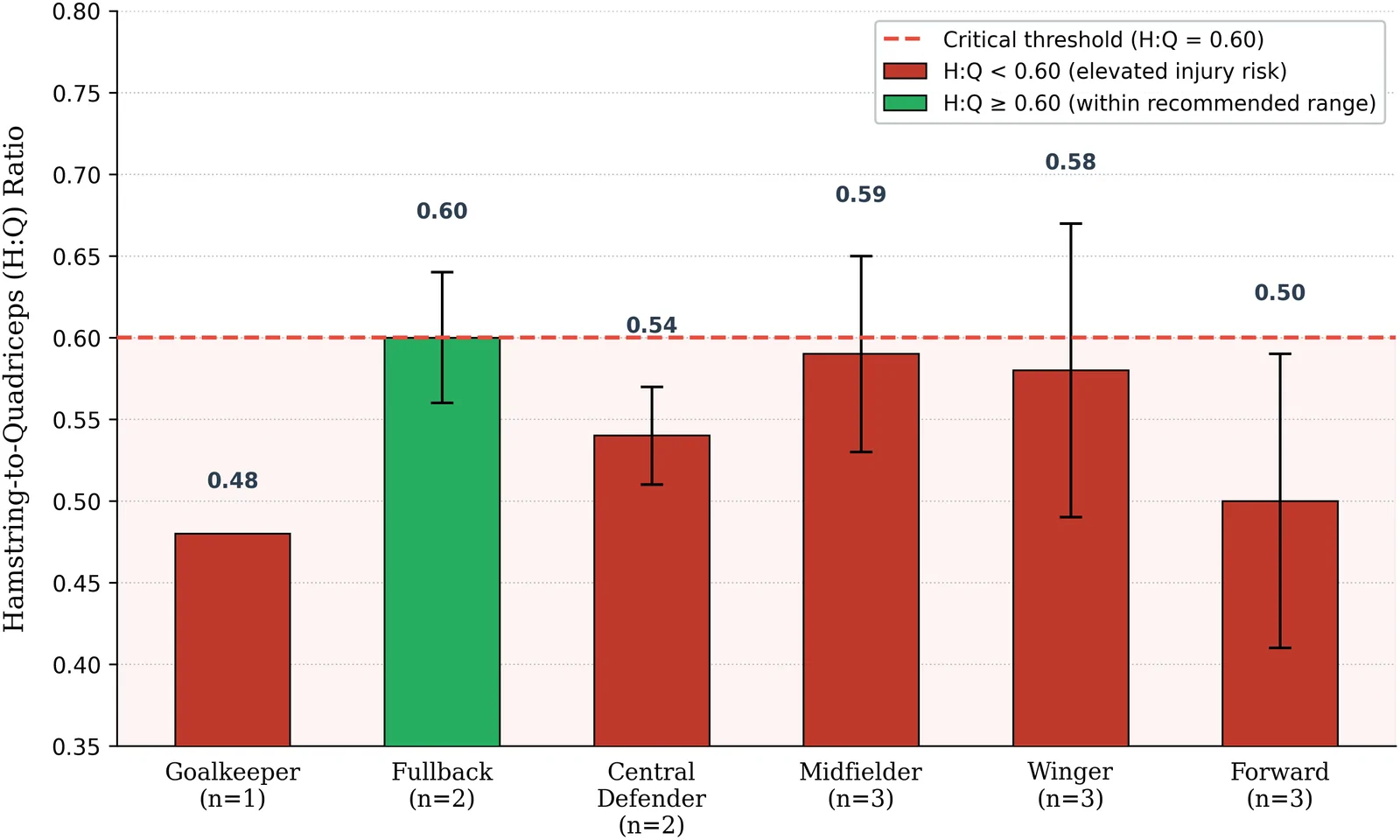
Positional differences in right knee extension strength, right-limb H:Q ratio, squat jump height, and countermovement jump height. Values are descriptive only because positional subgroup sizes were small and unequal.

Descriptive data by playing position are presented in Table 4 and illustrated in Figure 1. Subgroup sizes were small (n = 1–5 per position) and statistical between-group comparisons were therefore not performed. The data are presented for descriptive reference only, and observed differences between positional groups should not be interpreted as statistically significant or generalisable. Numerically, forwards presented the highest mean right knee extensor strength alongside the lowest mean right-limb H:Q ratio, midfielders presented the highest mean bilateral CMJ height, and the single goalkeeper presented an H:Q ratio below the 0.60 reference threshold. These observations are reported for completeness; given the very small positional subgroup sizes and the descriptive nature of the comparison, they should be interpreted as preliminary and exploratory only.

**Table 4:** Descriptive physical performance data by playing position (valid observations only).

Position	n	Age (years)	Ext R (kg)	H:Q R	SJ (cm)	CMJ (cm)
Goalkeeper	1	17	37.5	0.48	30.8	30.3
Fullback	5	20.4 ± 2.6	42.5 ± 1.3 (n = 2)	0.60 ± 0.04 (n = 2)	28.4 ± 3.6	28.9 ± 4.2
Central defender	2	20.5 ± 4.9	35.5 ± 4.6	0.54 ± 0.03	29.8 ± 7.8	26.9 ± 4.3
Midfielder	5	23.2 ± 5.5	37.2 ± 6.3 (n = 3)	0.59 ± 0.06 (n = 3)	28.8 ± 3.2	31.3 ± 3.7
Winger	3	22.3 ± 5.5	39.1 ± 2.3	0.58 ± 0.09	26.9 ± 7.1 (n = 2)	27.0 ± 4.5 (n = 2)
Forward	3	18.0 ± 2.6	41.8 ± 3.6	0.50 ± 0.09	33.0 ± 2.2 (n = 2)	29.1 ± 2.6 (n = 2)

Values are mean ± SD or single values where n = 1. Reduced n for some variables reflects missing strength or jump measurements. Ext R: maximal right knee extension force; H:Q R: right-limb hamstring-to-quadriceps ratio; SJ: squat jump; CMJ: countermovement jump.

## Discussion

This study provides a cross-sectional descriptive neuromuscular profile of elite Bulgarian female football players from a national-champion squad. The main findings were: (1) H:Q ratios below 0.60 were observed in 92.9% of assessed players (95% CI [68.5–98.7%]) on at least one limb; the right-limb mean was statistically below the 0.60 reference (p = 0.032, d = −0.64); (2) bilateral SJ and CMJ heights were nearly equivalent, producing a near-zero mean elasticity index that may indicate limited stretch-shortening cycle utilisation, although this construct was not directly assessed; (3) unilateral SJ and CMJ asymmetry exceeding 10% was observed in approximately half and one third of assessed players, respectively; and (4) descriptive positional differences were observed but were not formally tested and should be interpreted with caution given small subgroup sizes.

The high prevalence of H:Q ratios below 0.60 in the present cohort is consistent with previous reports of suboptimal hamstring-to-quadriceps balance in elite and sub-elite female football populations [15]. The H:Q ratio should not be interpreted as a direct injury-predictive marker in isolation, but rather as one of several modifiable neuromuscular characteristics that may inform individualised conditioning and screening strategies [6,7]. Importantly, a low H:Q ratio can arise from comparatively low hamstring strength, comparatively high quadriceps strength, or both; the present descriptive data do not distinguish between these patterns at the individual level. Conditioning interventions targeting H:Q balance should therefore consider hamstring strengthening (e.g., Nordic hamstring exercise, hip-hinge variants) as well as the broader role of quadriceps function in knee joint stability, deceleration, landing mechanics, and athletic performance [16]. Recommendations focused exclusively on hamstring conditioning would be a reductive interpretation of the present findings.

The positional pattern observed in the present cohort is presented descriptively. Numerically, forwards demonstrated the highest right knee extensor strength but the lowest right-limb H:Q ratio. While this pattern is broadly consistent with previous observations in female football positional profiling [15], the present subgroup size (n = 3 forwards) precludes any inferential generalisation. The same caveat applies to the observation that midfielders presented the highest bilateral CMJ height. These descriptive observations are best interpreted as hypothesis-generating for future, adequately powered positional comparisons in larger, multi-club samples.

The mean bilateral CMJ height of approximately 29 cm was lower than values commonly reported in elite female football cohorts from stronger international contexts [17,18]. This observation suggests scope for the development of explosive lower limb capacity within this squad, although direct cross-cohort comparisons are limited by differences in testing apparatus, jump protocol, instructional variation, and squad composition between studies. Further work involving multi-club Bulgarian samples and harmonised assessment protocols would help to establish normative reference values for the national context.

The near-zero mean elasticity index in the present cohort is consistent with limited augmentation of jump height through the countermovement at the group level. Caution is warranted in interpreting this descriptive observation. The elasticity index, while widely used as a practical surrogate marker, does not directly measure stretch-shortening cycle utilisation, eccentric-concentric coupling efficiency, or underlying neuromuscular contributors, which require force-time or kinetic analysis for direct assessment [13]. The substantial inter-individual variability observed (individual EI values ranged from approximately −21% to +19%) is therefore the more robust finding, and it indicates that group-level means may obscure clinically relevant individual differences. Practical inferences regarding plyometric capacity should be derived from individual rather than group-level EI values.

Unilateral jump asymmetry was also common within the cohort. More than half of the assessed players exceeded the 10% asymmetry threshold for SJ (52.9%, 95% CI [31.0–73.8%]) and approximately one third exceeded it for CMJ (35.3%, 95% CI [17.3–58.7%]). Previous research has emphasised that asymmetry magnitude varies by task and may be partly task-specific [19]. The right-limb directional dominance observed for both jump tasks in the present cohort is consistent with the predominance of right-footed players in the squad. Whether this magnitude of asymmetry is associated with prospective injury risk or performance decrement in elite female football remains a question for adequately powered longitudinal studies; the present data should not be over-interpreted in this regard.

Several limitations should be acknowledged. First, the sample was small (n = 19, with positional subgroups of n = 1–5) and was drawn from a single national-champion squad. This whole-squad sampling reflects the scope of the study and the absence of normative reference values for elite female football in this national context, but it limits both statistical power and the external generalisability of the findings to elite female football more broadly. Second, the cross-sectional design precludes any causal inference regarding the relationship between the neuromuscular characteristics identified and prospective injury risk or competitive performance; longitudinal injury surveillance is required to address this question. Third, isometric strength was assessed using a handheld dynamometer rather than the isokinetic dynamometry that remains the criterion standard for velocity-specific torque assessment [20,21]; consequently, the present strength values should not be interpreted as isokinetic-equivalent. Fourth, missing strength data for some participants (n = 5 for the strength battery) and missing jump data for some participants (n = 2 for the jump battery) reduced the effective sample size for certain analyses. Fifth, the elasticity index used here is a derived practical marker and does not directly capture stretch-shortening cycle utilisation. Sixth, habitual training load, menstrual cycle phase, and time of competitive season were not controlled or recorded, and may contribute to inter-individual variability in the present findings. Future studies should employ larger, multi-club samples with longitudinal injury surveillance, isokinetic dynamometry, and controlled assessment of contextual factors to establish the predictive validity of the screening profile presented here.

## Conclusions

Elite female football players from FC NSA Sofia Women, the Bulgarian national champions of the 2024/25 season, presented a high prevalence of H:Q ratios below 0.60 and frequent unilateral jump asymmetry above 10%. Mean bilateral SJ and CMJ heights were nearly equivalent, with substantial inter-individual variability in the elasticity index. Positional differences were observed only descriptively and should be interpreted with caution given the small subgroup sizes. From a practical perspective, these findings support the inclusion of routine pre-season neuromuscular screening combining bilateral isometric knee strength testing and unilateral jump assessment as part of individualised conditioning and monitoring programmes in elite Bulgarian women’s football. Conditioning interventions should address both hamstring and quadriceps capacity, rather than focusing on hamstring strengthening in isolation, and should be guided by individual rather than group-level data. The data presented here may serve as a preliminary descriptive reference for elite female football in the Bulgarian national context, pending replication in larger, multi-club, longitudinal studies.

## Supporting information

S1 Dataset De-identified individual-participant data underlying the descriptive and inferential analyses reported in the manuscript (Microsoft Excel format). The file contains three sheets: a README sheet describing anonymization procedures and ethics context; a Data dictionary sheet defining all variables, units, and computed indices; and an Anonymized data sheet containing the participant-level data (n = 19). Personal names and exact ages have been removed; ages are reported in 5-year bands; and Participant IDs (P01–P19) have been randomly reassigned and are unrelated to the original recruitment order. No additional identifying information is included.
(XLSX)
